# The neurochemistry of learning-driven sensory eye dominance plasticity

**DOI:** 10.1162/imag_a_00237

**Published:** 2024-07-22

**Authors:** Ka Yee Kam, Dorita H.F. Chang

**Affiliations:** Department of Psychology, The University of Hong Kong, Hong Kong, China; The State Key Laboratory of Brain and Cognitive Sciences, The University of Hong Kong, Hong Kong, China

**Keywords:** sensory eye dominance, perceptual learning, plasticity, magnetic resonance spectroscopy, GABA

## Abstract

Sensory eye dominance (SED) refers to a functional asymmetry of the two eyes that is thought to result from the visual cortex assigning uneven weighting to the two eyes’ data. Dichoptic perceptual training has been shown to improve (reduce) SED in visually normal individuals, with behavioral improvements accompanied by alterations of neural responses in the primary visual cortex. The mechanisms underlying these learning-driven neural changes are not well understood. Here, using magnetic resonance spectroscopy, we determined how inhibitory mechanisms in the early visual cortex (EVC) govern SED plasticity by measuring γ-aminobutyric acid (GABA) concentration changes before and after perceptual training. Fifty normal-sighted observers were trained on a dichoptic or binocular variant of a signal-in-noise (left–right) motion discrimination task. We observed significant shifts in SED following dichoptic (but not binocular) training. Before training, both groups exhibited lower GABA concentrations in the EVC when signals were presented to the dominant eye. Only after dichoptic training, GABA concentrations in the EVC increased during presentations of signals to the dominant eye and decreased during presentations of signals to the non-dominant eye. Our data suggest that dichoptic training drives changes in SED by promoting a rebalancing of interocular inhibition in the EVC.

## 1 Introduction

An optimal binocular visual system requires a sensory balance in which neither eye dominates the other. When one eye makes a greater weighted contribution to the binocular neural network, such sensory eye dominance (SED) may impair binocular visual processing, especially in cases of severe dominance. While exaggerated SED is a prominent feature among certain clinical groups, such as patients with amblyopia (Ding et al., 2013;Zhou, Clavagnier, et al., 2013), a milder degree is commonly observed in the visually normal population (Bossi et al., 2018;Handa et al., 2012;Li et al., 2010;Yang et al., 2010;Zhang et al., 2011). Encouragingly, an increasing body of research has shown that mechanisms governing SED in human adults retain a certain degree of plasticity (Bankó et al., 2022;Elhusseiny et al., 2021;Hess et al., 2010a;Hou & Nicholas, 2022;Tuna et al., 2020;Zhou et al., 2014). In particular, training protocols that adopt dichoptic presentation of motion-in-noise stimuli have been proven effective in altering SED in visually normal and impaired individuals (Hess et al., 2010a;Kam & Chang, 2021). By presenting signal dots and noise dots to separate eyes, this approach creates viewing conditions that demand the extraction of motion signals while simultaneously filtering out noises originating from the other eye. Training under these conditions encourages cooperative reconciliation of data presented to both eyes, as the task cannot be solved monocularly. Considering the recent advancements in protocols designed to enhance plasticity in sensory eye balance, it is of both scientific and clinical importance to unravel the neural substrates that underlie SED plasticity, particularly those induced by dichoptic perceptual training.

Binocular vision relies on a complex interplay of excitatory and inhibitory interactions between the inputs from the two eyes. Models of binocular integration propose that the visual system regulates the relative strength of input from each eye through gain control mechanisms that function both before and after excitatory binocular summation (Meese et al., 2006). The proposed pre-summation gain-control mechanism, in particular, involves contralateral inhibitory interactions between the two eyes. To put it differently, a balanced binocular system is maintained, at least in part, by approximately equivalent reciprocal inhibition between the anatomically segregated inputs from each eye to V1. Any asymmetry in interocular inhibition may thus contribute to SED (Huang et al., 2010;Sengpiel et al., 1994). This theoretical model aligns with the extensively documented data from amblyopic visual systems, which indicate asymmetric interocular inhibition, with the dominant eye exerting stronger suppression over the amblyopic eye than vice versa (Ding et al., 2013;Harrad et al., 1996;Huang et al., 2011;Sengpiel & Blakemore, 1996;Zhou et al., 2018). Similarly, individuals with clinically normal vision have been observed to exhibit an imbalance of interocular inhibition. For example, when tested on a dichoptic signal-in-noise motion task, visually normal observers showed varying task performance depending on the eye to which signals were presented (García-Pérez & Peli, 2019;Kam & Chang, 2021;Li et al., 2010), implying that the two eyes differ in their effectiveness in suppressing noise from the other eye. Further, during binocular rivalry, normal-sighted observers showed varying durations and frequencies of perceptual dominance for each eye (Dieter et al., 2017;Xu et al., 2011), reflecting asymmetrical interocular interactions. In this vein, then, learning-driven changes in SED could result from a readjustment of interocular inhibition. But, where and what are the mechanisms that account for this readjustment of interocular inhibition?

To probe the sites and mechanisms of learning-driven improvement in SED, we previously measured functional magnetic resonance imaging (fMRI) responses before and after dichoptic signal-in-noise motion training (Kam & Chang, 2023). We found that learning-induced changes in SED were accompanied by alterations of (the pattern of) neural responses in the primary visual cortex (V1). Specifically, responses of V1 predicted SED before training, with different patterns of responses observed when signals were presented to the dominant versus the non-dominant eye. These differences were no longer evident after dichoptic perceptual training. These results suggest that dichoptic training alters SED by potentially driving a reweighting of input from the two eyes in V1. Building upon earlier discussions on binocular integration and the well-documented understanding that the effective extraction of signal from noise presented to different eyes entails not only a simple summation of excitatory monocular inputs but also the involvement of interocular inhibitory mechanisms (Hess et al., 2007;Zhou, Huang, et al., 2013), we speculate that the observed changes in the relevance of V1 responses to eye dominance following dichoptic perceptual training may reflect a rebalancing of interocular inhibition. This could presumably be a result of a weakening of inhibition originating from the dominant eye, and/or a strengthening of inhibition originating from the non-dominant eye.

Emerging evidence indicates that visual cortical interocular suppression originates from inhibitory interactions mediated by gamma-aminobutyric acid (GABA). GABA is the primary inhibitory neurotransmitter in the brain and plays a pivotal role in shaping visual plasticity (Frangou et al., 2018,^2019^;Sale et al., 2010). Animal pharmacological studies demonstrated that applying GABA receptor antagonists to V1 in amblyopic cats and rats significantly reduced binocular suppression and improved visual function in the amblyopic eye (Sengpiel et al., 2006;Vetencourt et al., 2008). Correspondingly, in human amblyopes, lower levels of visual cortical GABA have been observed in conjunction with weaker suppression of the fellow eye by the amblyopic eye (Mukerji et al., 2022). Studies testing visually normal adults on a binocular rivalry task have shown that individuals with higher GABA concentrations in the visual cortex tended to experience greater perceptual suppression (Mentch et al., 2019;Robertson et al., 2016), and pharmacologically augmenting GABA concentration with lorazepam further strengthens perceptual suppression during rivalry (van Loon et al., 2013).

While the evidence discussed thus far has predominantly centered on the connection between GABA and interocular suppression, a recent magnetic resonance spectroscopy (MRS) study explored the link between GABA and SED in normal vision. By monocularly stimulating either the dominant or the non-dominant eye, it has been demonstrated that individuals with stronger eye dominance tended to exhibit a greater interocular difference in GABAergic inhibition in V1, with higher GABA levels during viewing with the dominant eye (Ip et al., 2020). Of greater relevance to the present work, GABA has also been implicated in SED plasticity.Lunghi et al. (2015)measured GABA levels before and after short-term monocular deprivation and observed a reduction in*resting*GABA concentration in V1 after monocular deprivation, with the extent of GABA reduction correlating with changes in SED. However, they observed no significant changes in the GABA levels during monocular or binocular active viewing. While this study provides the first evidence of an association between GABAergic inhibition and SED plasticity in humans, it offers limited insight into how the changes in GABA related to the functional product of SED—binocular vision itself—since these changes were not observed during visual stimulation. Additionally, it is unclear as to whether longer-term dichoptic perceptual training produces mechanistically-comparable (GABAergic changes) in V1, or whether such training produces a distinct set of neurochemical changes.

Here, we used MRS to ask how inhibitory mechanisms in the early visual cortex (EVC, which includes V1), as measured by metabolite concentrations of GABA, mediate learning-driven plasticity in sensory eye balance. We measured GABA concentrations in the EVC and motor cortex (M1) before and after five days of dichoptic signal-in-noise motion training and compared the resultant behavioral and neurochemical changes with those of a group trained on a binocular variant of the same task (signal and noise dots presented to both eyes). The choice of signal-in-noise motion stimuli was based on its established efficacy in reducing SED in both clinical and normal populations (Hess et al., 2010b;Kam & Chang, 2021,^2023^), as well as our previous findings indicating that training with dichoptic signal-in-noise motion stimuli modulated pattern responses within V1 (Kam & Chang, 2023). We elected to measure GABA concentration in the M1 as a control to ensure that any changes in GABA levels after training were specific to the EVC and not indicative of global changes or motor-training-related changes (i.e., button presses during perceptual training).

Based on previous work, we expected that training on the dichoptic, but not the binocular variant of the signal-in-noise motion task, would lead to behavioral shifts in eye dominance. We reasoned that unlike for dichoptic viewing, which requires the extraction of signal from noise presented to separate eyes, the binocular variant does not demand the same level of cooperative reconciliation of data presented to both eyes as the task could be solved on a purely monocular basis, with signal and noise presented equally to both eyes. Therefore, training under dichoptic presentation should be more effective in engaging and altering SED-relevant mechanisms. We further predicted that if noise-based dichoptic training acted on visual inhibitory mechanisms, any changes in sensory eye balance resulting from dichoptic perceptual training would be accompanied by corresponding changes in GABA concentrations in the EVC. By contrast, we anticipated that no changes would be observed in GABA concentrations in M1 following either type of training.

## 2 Material and Methods

### 2.1 Participants

A total of 50 observers were recruited for this study (mean age 22.4 ± 3.02 years, 28 females). All observers had normal or corrected-to-normal vision, including normal binocular fusion, as confirmed by the logMAR chart and the Worth-4-dots test, and were screened for MRI contraindications. All observers provided written informed consent in accordance with the ethical review approved by the Human Research Ethics Committee of The University of Hong Kong. Observers were randomly assigned to two training groups, with one group receiving training on a dichoptic variant of the signal-in-noise motion task (N = 25, mean age 22.2 ± 3.63 years, 14 females), and the other on a binocular variant of the same task (N = 25, mean age 22.6 ± 2.30 years, 14 females). We excluded three observers (two from the dichoptic training group and one from the binocular training group) from the final analysis due to contamination of the spectra data.

### 2.2 General procedure

**Fig. 1.**
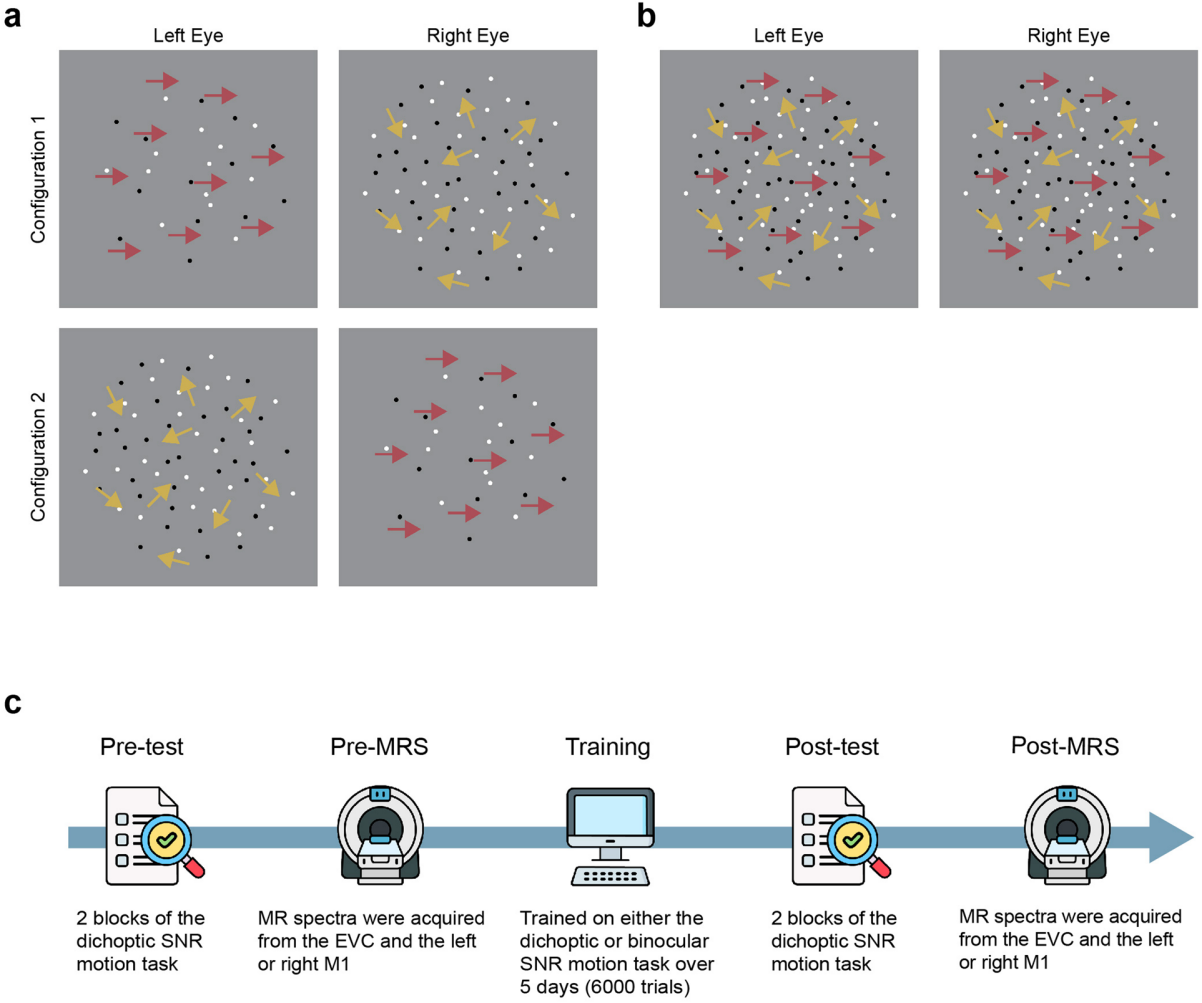
Schematics of a dichoptic and binocular variant of the signal-in-noise motion task and the general experimental procedure. (a) The dichoptic variant involved presenting signal and noise dots to different eyes on each trial. Two stimulus configurations were used such that we randomly presented signal dots to either the left (configuration 1) or the right eye (configuration 2) on each trial. (b) The binocular variant presented signal and noise dots to both eyes on each trial. (c) The general experimental procedure.

Figure 1cpresents a schematic overview of the experimental procedure. Before and following perceptual training, both groups completed laboratory tests and an MRS scan. During both the laboratory test and MRS scans, participants were tested on the dichoptic signal-in-noise motion task. The two groups received perceptual training on either a dichoptic or binocular variant of the signal-in-noise motion task for one hour per day over five consecutive days (6000 trials). The post-training laboratory test was administered immediately following the last training block, while the post-training MRS scan was conducted on the day after the last training session.

### 2.3 In-laboratory testing and training

#### 2.3.1 Apparatus

Visual stimuli were generated using custom software written in MATLAB with extensions from Psychtoolbox (Brainard, 1997). Stimuli were presented dichoptically using a shutter-presentation setup that utilized a 120 Hz ASUS 3D-vision ready LCD (resolution: 1920 x 1080 pixels) and NVIDIA 3D Vision 2 shutter goggles. Prior to the start of each experimental session, different geometric test patterns were presented independently to each eye, and the observers were required to report the perception of each eye. There were no reported incidents of crosstalk between eyes. In order to maintain a constant viewing distance and head position throughout the experiment, observers were required to rest their heads on a chinrest that was secured at a fixed distance of 50 cm from the display.

#### 2.3.2 Tasks and stimuli

The stimulus was displayed on a uniform gray background enveloped by a binocularly presented frame of solid black and white squares. This grid-like frame was designed to provide an unambiguous background reference, thereby facilitating binocular fusion. The in-laboratory dichoptic signal-in-noise motion stimuli consisted of an equivalent number of black and white dots, each with a size of 0.2 deg. The dots moved within a central aperture of 9 deg in diameter, with a velocity of 2 deg/s, and with no limited lifetime. At the beginning of each trial, the position of each dot was randomly assigned within the aperture. In the event that any dots had an impending collision or were to move outside the aperture on the next frame, they were redrawn to a new random position.

On each trial, signal dots carrying a coherent motion direction (either left or right) were presented to one eye, while noise dots carrying random motion directions were presented to the alternate eye. Observers were instructed to make a two-alternative force choice judgment by pressing the left or the right arrow keys on the keyboard to indicate the net motion direction of the dots. We manipulated task difficulty by varying the signal-to-noise ratio (SNR) from 0 to 100 on each trial using QUEST staircase procedure sampling thresholds at an 82% correctness level. The detectability of motion direction was dependent on the percentage of signal dots that moved coherently in a single direction. A block of trials consisted of two interleaved staircases of 60 trials each that randomly presented signal dots to either the left (Fig. 1a, configuration 1) or the right eye (Fig. 1a, configuration 2), resulting in a total of 120 trials for one test block. Stimuli were presented for a duration of 500 ms, followed by a response period of 1000 ms, and a fixed interstimulus interval of 300 ms. Prior to the pre-test, observers completed a brief practice session consisting of 20 trials to familiarize themselves with the task. All the observers completed two test blocks in both pre- and post-training laboratory tests. All stimulus and task parameters employed in the training blocks remained consistent with those used in the pre- and post-training test blocks, with the exception that auditory feedback was provided during the training blocks but not during the test blocks.

For the binocular variant of the signal-in-noise motion task, all stimulus and task parameters were identical to those outlined earlier for the dichoptic variant, with the sole distinction being that the signal and noise dots were presented to both eyes on each trial (Fig. 1b). The binocular variant was employed as a training task only and was not administered in pre- or post-training tests.

### 2.4 MRS acquisition, design, and analysis

#### 2.4.1 Apparatus

Dichoptic presentation of visual stimuli in the magnet was achieved via a ProPixx projector (resolution of 1920 x 1080 pixels; refresh of 120 Hz) equipped with a DepthQ circular polarizer. Images were projected to a standing mirror placed at a 45° angle behind the bore, which, in turn, projected onto a translucent 3D rear projection screen positioned at the base of the bore. Observers viewed the stimuli through a 45° tilted mirror mounted above the head coil, with passive polarized filters inserted in the custom MR-compatible frames. A second optional corrective lens was also inserted when necessary to correct for myopia.

#### 2.4.2 Tasks and stimuli

MR spectra were acquired while observers were completing the dichoptic signal-in-noise motion task. The dichoptic signal-in-noise motion stimuli used in the bore were the same as those used in the laboratory, with the exception of two changes: First, both signal and noise dots were presented at 70% contrast. Second, the diameter of the stimuli aperture was set to 10 deg, with each dot subtending 0.22 deg and moving with a velocity of 4 deg/s. These alterations were introduced to amplify the stimulus while minimizing crosstalk in the bore, mitigating the challenges of performing visual tasks in a confined space.

#### 2.4.3 MRS acquisition

Magnetic resonance data were acquired using a GE SIGNA Premier 3.0T scanner with a 48-channel head coil. For each participant, a high-resolution T1-weighted anatomical image (MPRAGE, TR = 7 ms; TE = 2.8 ms; FOV = 256 x 256, flip angle = 8°) was acquired at a spatial resolution of 1 mm^3^to permit accurate placement of the region-of-interest (ROI, voxel). Single-voxel MRS data were acquired using a MEGA-PRESS sequence with the following parameters: TR = 2000 ms, TE = 68 ms, 256 transients, spectral bandwidth = 5000 Hz, and 4096 data points. Two interleaved datasets were collected during the scan, in which the editing pulses (14 ms 180° Gaussian-weighted sinc pulses) were applied at 1.9 ppm (edit-ON) and 7.5 ppm (edit-OFF), respectively. Before each MRS scan, volumes were shimmed to optimize magnetic field homogeneity. The acquisition time for a single MRS scan was 9 minutes and 20 seconds.

**Fig. 2.**
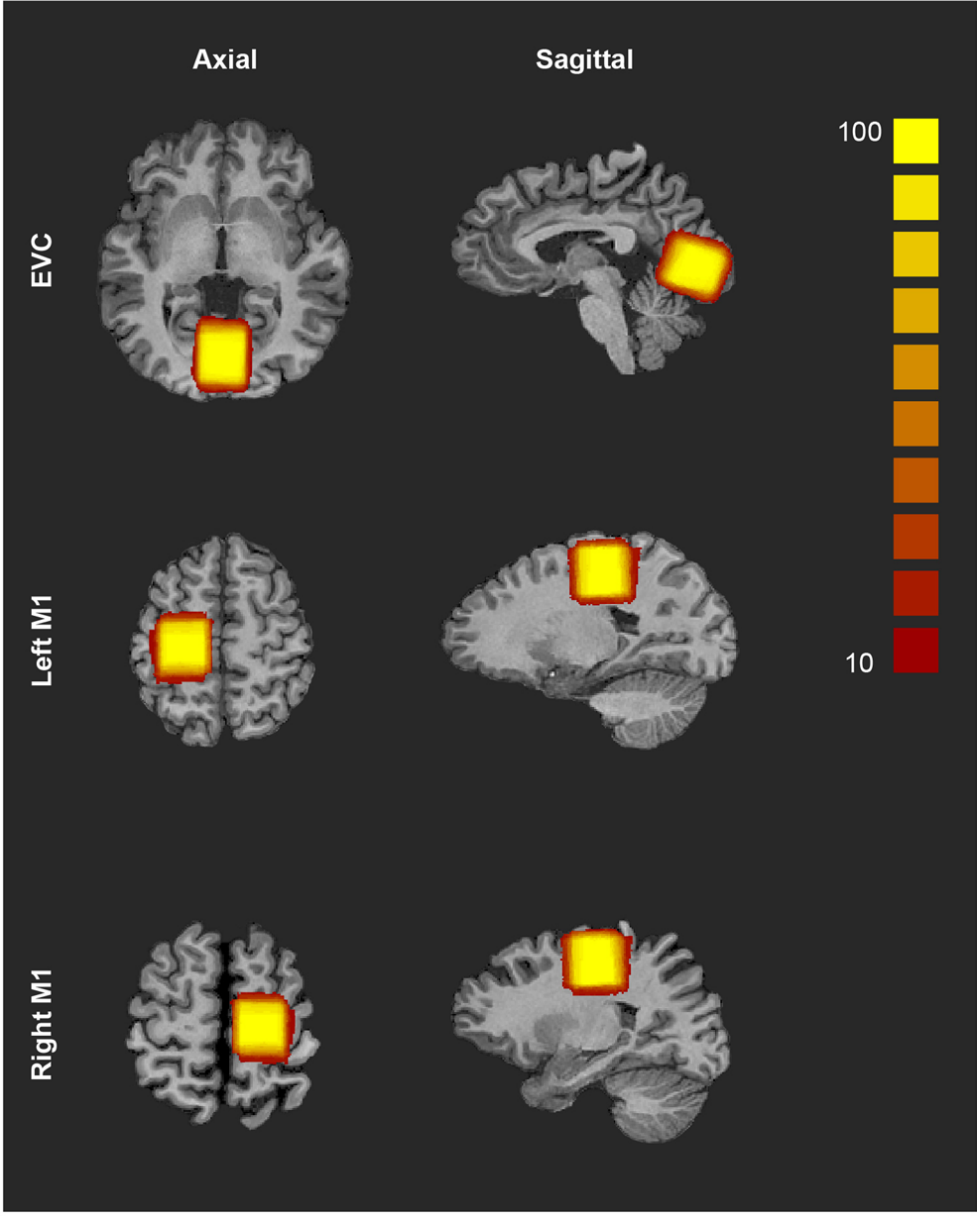
MRS voxel probability map. The early visual cortex (EVC) voxel was centered bilaterally on the calcarine sulcus, covering V1 in both hemispheres. The left/right motor cortex (M1) voxel was positioned on the left/right precentral gyrus anterior to the central sulcus. The color bar indicates the probability of voxel overlap across participants for MRS voxels present in at least 10% of the participant’s MRS masks.

MR spectra were acquired from the early visual cortex (EVC) and left/right motor cortex (M1), with a voxel size of 3 × 3 × 3 cm.Figure 2provides an illustration of voxel locations and the probability of voxel overlap across participants. The EVC voxel was centred bilaterally on the calcarine sulcus to include as much V1 as practically feasible in both hemispheres and was carefully oriented to avoid placements that might bring about excessive lipid contamination. Considering the unique brain morphology of each individual, efforts were made to minimize potential contact with the sagittal sinus and tentorium. Nevertheless, in situations where unavoidable contact occurred, it is important to note that this would have impacted the signal-to-noise ratio but not the overall results as such artefacts would have been present consistently in both stimulus conditions where motion signal was presented to either eye, and both before and after training. While the voxel placement was primarily intended to target bilateral V1, the cubic shape and dimension of the voxel resulted in the inclusion of certain portions of V2 and minor contact with V3. The left/right M1 voxels were positioned on the left/right precentral gyrus anterior to the central sulcus and oriented to avoid the midline of the brain. For each participant, MRS data were acquired at the EVC and either the left or right M1. To control for potential confounding factors, the hemisphere of the M1 being imaged was randomized. Spectra were acquired from the left M1 for 13 observers from the dichoptic training group and 12 from the binocular training group. The mean Talairach and MNI coordinates of the voxel’s centroid for EVC and left/right M1 are presented inTable S1.

To ensure consistent voxel placement across sessions and between participants, the placement of the ROI was guided by high-resolution T1 structural MRI images and manually positioned on the anatomical landmarks of each individual. To verify that the voxel position was similar across sessions and between participants, we conducted 2 (Session–Pre/Post) × 2 (Group–Dichoptic/Binocular) mixed ANOVAs, separately for the coordinates of each of the EVC, left M1, and right M1 voxels. The analyses revealed no significant main effects or interactions between Session and Group for any of the ROIs, indicating that the voxel position was consistent across sessions and between groups.

#### 2.4.4 Design and procedure

Before commencing MRS data acquisition, we obtained individually tailored stimulus test values for the main experimental runs by having each participant complete a single behavior-only run of the dichoptic signal-in-noise motion task while lying inside the bore. Similar to the in-laboratory tests, one run consisted of two interleaved staircases of 60 trials, each corresponding to the two stimulus configurations (signals dots presented to the left or the right eye). We calculated the mean test values for subsequent image acquisition by averaging the last 30 trials for each configuration and defined a range of stimulus values of ±1 standard deviation from this mean value. We then sampled the signal-to-noise ratio from this range for each trial. This approach ensured that task difficulty was matched across participants and stimulus configurations.

MR data acquisition began with a T1-weighted MPRAGE. Subsequently, MR spectra were acquired from the two ROIs–EVC and either the left or right M1, concurrently while participants performed the dichoptic signal-in-noise motion task. Two MRS acquisition runs were obtained for each ROI, with one run presenting the signal dots to the left eye and the other presenting the signal dots to the right eye. This resulted in a total of four MRS acquisition runs in both pre- and post-training scans. The order of ROI spectra acquisition and stimulus configuration presentation was randomized and counterbalanced across participants to minimize potential order effects. In a particular MRS run, stimuli were presented in a block design, with each block lasting 16 s. This was done not for the purpose of eventual block-segregated analyses but to maintain consistency with our previous fMRI work (Kam & Chang, 2023). Each run comprised two block types: stimulus blocks and fixation blocks. Each stimulus block consisted of eight trials, each presenting the stimulus for 500 ms followed by a 1500 ms response period. During the response period, observers were asked to judge the net motion direction of the dots by pressing buttons on the response box. A fixation block contained a white fixation cross with a size of 0.8 deg, presented at the center of the screen for 16 s. The stimulus blocks were interleaved with fixation blocks. A full scan session lasted approximately 65 minutes.

### 2.5 Data analysis

#### 2.5.1 Behavior

In accordance with our earlier work (Kam & Chang, 2021,^2023^), SED was quantified by deriving a binocular balance index using the dichoptic signal-in-noise motion test task. The binocular balance index was computed using the following formula: (*E*_weak_-*E*_strong_)/(*E*_weak_+*E*_strong_), where*E*_weak_and*E*_strong_represent the thresholds obtained when the signal dots were presented to the non-dominant and dominant eyes, respectively. An index of zero indicates an absence of eye dominance. Conversely, a deviation from zero suggests the degree of eye dominance, with a greater deviation indicating stronger eye dominance. For each participant, the dominant eye was determined in the pre-test. Any degree of dominance yielded a positive binocular balance index. An index value closer to zero following training would indicate a decrease in eye dominance, and any negative index value would signify a change in the dominant eye.

#### 2.5.2 MRS data analysis

MRS data were processed and analyzed in MATLAB using Osprey toolbox version 2.4.0 (Oeltzschner et al., 2020). To ensure the quality and suitability of the acquired spectra for further analysis, we applied a series of pre-processing steps (Choi et al., 2021). First, the raw spectra data were eddy current-corrected using the Klose method (Klose, 1990). Next, individual transients were aligned separately within the edit-ON and edit-OFF conditions by using a robust spectra registration algorithm (Mikkelsen et al., 2020). To generate the final difference spectra for quantification, the weighted-averaged edit-ON and edit-OFF spectra were subsequently aligned by optimizing the relative frequency and phase to minimize the water signal in the difference spectrum. The final difference spectra were obtained by subtracting the edit-OFF from the edit-ON spectra. Finally, a Hankel singular value decomposition (HSVD) filter (Barkhuijsen et al., 1987) was applied to remove any residual water signal between 4.2 and 4.9 ppm. Example spectra obtained from the EVC and the M1 voxel are presented inFigure S4.

To ensure the reliability and accuracy of subsequent analyses, we inspected the quality of the acquired MRS data. First, data were visually inspected for noise, voxel misplacement, and lipid contamination. Two participants, one from each training group, were excluded from further analyses due to significant lipid contamination (the largest peak in the edit-OFF spectrum was observed at a resonance frequency different from 2 ppm) in at least one of the acquired spectra. Second, the quality of the included spectra was evaluated based on linewidth and signal-to-noise ratio (SNR). The linewidth was calculated as the full-width half-maximum of a single-Lorentzian fit to the N-acetylaspartate (NAA) peak in the spectral range between 1.8 and 2.2 ppm. The mean linewidth for the EVC voxel was 7 Hz before and after training, while for the M1 voxel, it was 6.2 Hz and 6.3 Hz before and after training, respectively. SNR was determined as the ratio between the amplitude of the NAA peak and the standard deviation of the detrended noise in the spectral range between -2 and 0 ppm. One observer from the dichoptic training group was excluded from further analyses due to unusually low SNR in one of the acquired spectra (deviated by more than three standard deviations from the mean SNR). The average SNR for the EVC voxel was 115, before and after training. For the M1 voxel, the average SNR before and after training were 127 and 130, respectively. The linewidth and SNR were consistent across scanning sessions for both locations and were within the range of the previously reported linewidth and SNR from a large-scale analysis of multi-vendor (including GE), multi-site single-voxel GABA-edited data (Craven et al., 2022).

Metabolite estimates were determined using Osprey’s frequency-domain linear combination model, with a model range of 0.5 to 4 ppm to include all signals in the GABA-edited difference spectrum and a sparse spline knot spacing of 0.55 ppm. These parameters were chosen based on the recommendations fromZöllner et al. (2022). The residuals of the model fit for the EVC voxel were 13.6 and 13.9 before and after training, respectively, while for the M1 voxel, the residuals were 16.6 and 16.8 before and after training, respectively. Given that MRS measurements of GABA with MEGA-PRESS sequence are known to be contaminated by co-edited macromolecules (MM_3co_), we included a parametrized Gaussian MM_3co_basis function in the model. This strategy enabled the model to account for the co-edited MM signals, improving the specificity of GABA measurements. However, it is important to note that the MM signals were not completely separated and eliminated from the spectra. Therefore, we refer to the estimated concentration of GABA as GABA+, representing the combined amplitude estimates for both GABA and MM_3co_. The reported GABA+ concentration was alpha-corrected (Harris et al., 2015) to account for the known variation in GABA levels across different tissue types, with higher levels in the gray matter than in the white matter (Jensen et al., 2005). In addition, the concentration was normalized with respect to the concentration of water in the brain, ensuring that the reported GABA+ concentration was referenced to a common internal standard.

Before conducting any statistical analyses, we normalized GABA+ data to the individual regional mean of GABA+ concentrations using the formula:$\frac{{\text{GABA+}}_{\text{DE/NDE}}}{{\text{GABA+}}_{\text{regional mean}}}$, where GABA+_regional mean_represents the individual mean of GABA+ concentration for each ROI, calculated by averaging the concentration values obtained from the two MRS acquisition runs within each session (i.e., the mean of GABA+ concentration obtained when the signal dots were presented to the dominant and non-dominant eye) and GABA+_DE/NDE_represents the concentration value obtained when the signal dots were presented to the dominant or non-dominant eye. This normalization was performed to account for individual differences in GABA+ levels and to reduce variability in the data due to differences in GABA+ levels across brain regions.

## 3 Results

### 3.1 Behavior

**Fig. 3.**
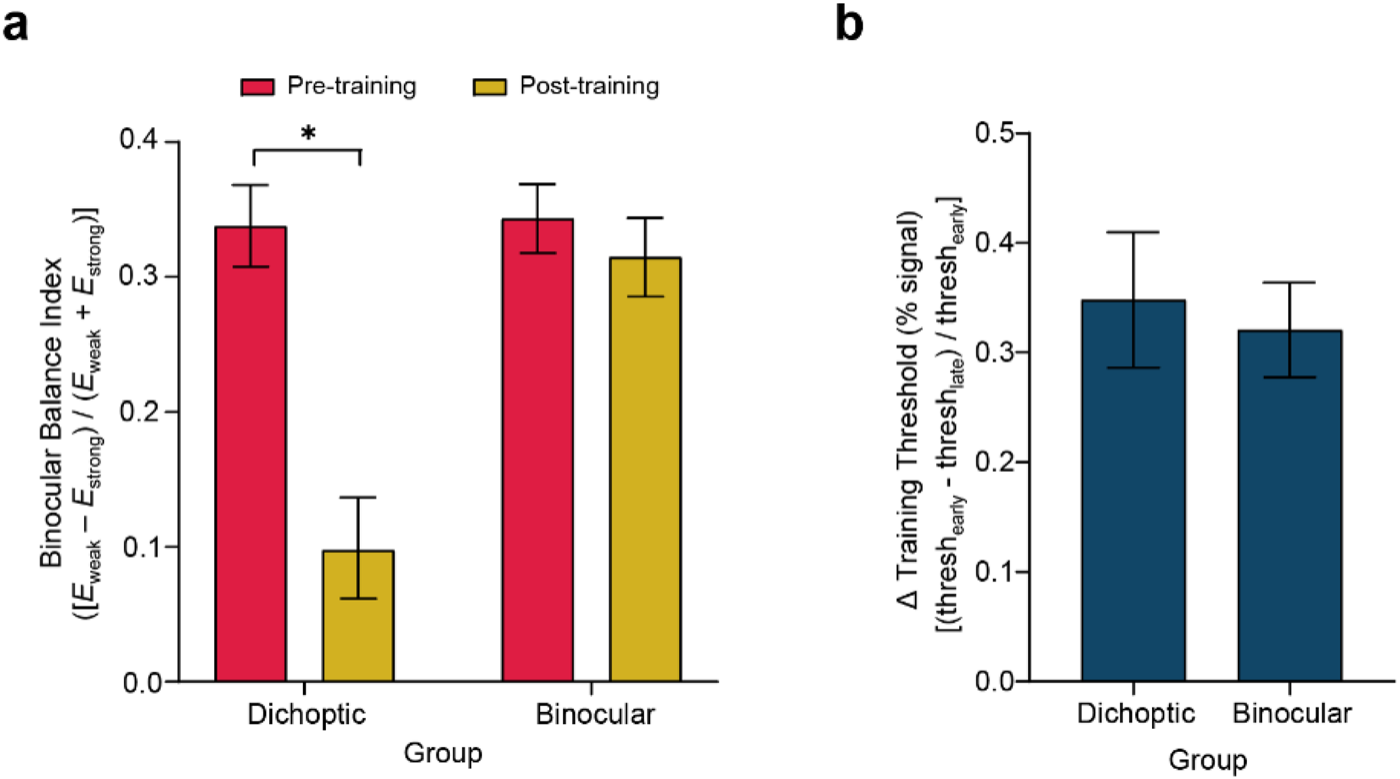
Behavioral results illustrating the changes in sensory eye dominance and the degree of learning. (a) Sensory eye dominance in the pre- and post-test for the dichoptic (N = 23) and binocular (N = 24) training groups, as indexed by the binocular balance index derived from the dichoptic signal-in-noise motion test task. An index of zero denotes no dominance. (b) Degree of learning achieved by each training group. For each participant, training threshold changes for the respective training tasks were calculated by [(thresh_early_–thresh_late_)/thresh_early_], where thresh_early_and thresh_late_were derived by averaging the thresholds obtained from the first and the last three training blocks, respectively. Error bars represent ±1 SEM. *p < 0.05.

First, we examined changes in SED following the completion of the five-day perceptual training protocol. Aligning with our previous findings (Kam & Chang, 2023), significant changes in SED were observed among observers who received training on the dichoptic signal-in-noise motion stimuli. No changes in SED were observed in the binocular training group, despite comparable levels of learning effectiveness between the two groups. Specifically, the effect of perceptual training on binocular balance index was tested across training groups and testing sessions by means of a 2 (Group—dichoptic/binocular) × 2 (Session—before/after training) mixed ANOVA. The results revealed a significant interaction between training group and session (F_(1, 45)_= 22.10, p < 0.001, η^2^_p_= 0.329,Fig. 3a). Follow-up Bonferonni-corrected comparisons indicated that SED reduced post- versus pre-training, but only for those who were trained on the dichoptic signal-in-noise motion stimuli (t_(22)_= 6.05, p < 0.001). By contrast, the binocular training group exhibited no significant changes in SED after training (t_(23)_= 1.259, p = 0.221).

To evaluate the degree of learning achieved by each training group, we compared their performance during the “early” and “late” training stages of their dedicated training task. For each participant, we calculated task performance for the early (thresh_early_) and late (thresh_late_) training stages by averaging the motion coherence thresholds obtained from the first three and last three training blocks, respectively. We then conducted Bonferonni-corrected paired t-tests independently for the two training groups. The results indicated significant improvements in their dedicated training task for both the dichoptically (t_(22)_= 5.17, p < 0.001) and binocularly (t_(23)_= 5.86, p < 0.001) trained groups.

Subsequently, we conducted two analyses to compare learning effectiveness between the two training groups. First, we compared training threshold changes for the respective training tasks (computed as [(thresh_early_–thresh_late_)/thresh_early_]) and conducted a between-group comparison using an independent t-test. Un-normalized thresholds are presented inFigure S1. Our analysis revealed no significant differences between the two groups in terms of percentage threshold changes (t_(45)_= 0.365, p = 0.717,Fig. 3b). Second, we used a signal-parameter logarithmic functionb=kln(a), whereaandbrepresent the training block and motion threshold, respectively, to fit the training data of each observer individually to estimate their learning rate (k). The learning rate parameter was not significantly different between the two training groups (t_(45)_= -1.648, p = 0.106). Collectively, our data indicated that both training tasks were effective in enhancing performance on the trained task and that the learning effectiveness was comparable between the dichoptic and binocular training groups. As such, it is unlikely that the observed reduction in SED in the dichoptic (but not binocular) training group can be attributed to more efficient training in this group versus the other.

### 3.2 MRS data

Before perceptual training, our MRS data showed clear differences in GABA+ concentrations in the EVC, with higher GABA+ concentrations detected when signals were presented to the non-dominant eye versus when they were presented to the dominant eye. This was true for both groups. Following dichoptic perceptual training, GABA+ concentrations decreased during presentations of signals to the non-dominant eye and increased during presentations of signals to the dominant eye (relative to pre-training). By contrast, GABA+ concentrations in the EVC for those trained on the binocular variant of the signal-in-noise motion task did not change after training. While GABA+ levels in the EVC varied depending on which eye received the signals and changed with dichoptic perceptual training, GABA+ levels in the M1 region did not show such variations or learning-related changes.

**Fig. 4.**
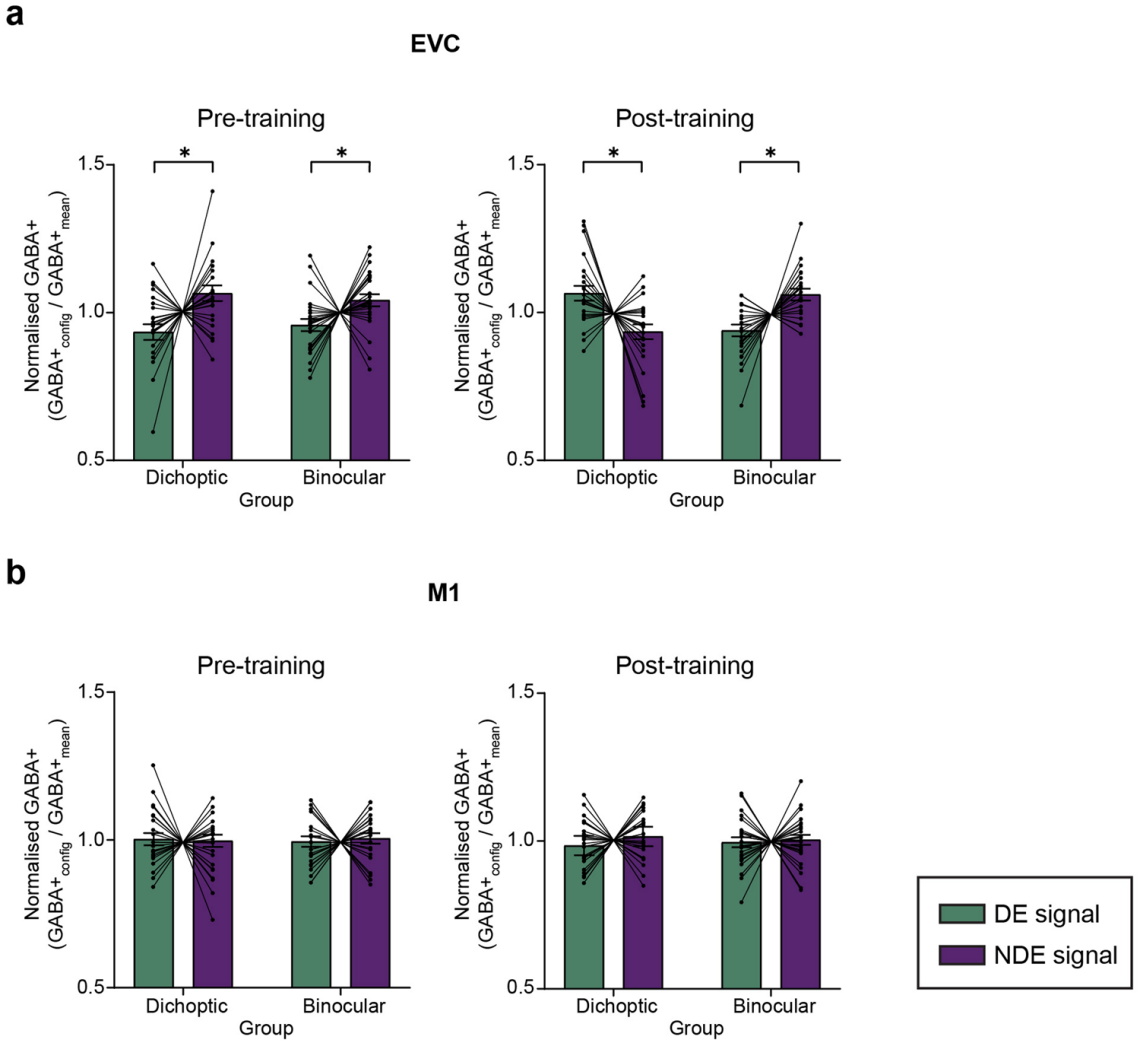
MR spectroscopy measurements of γ-aminobutyric acid (GABA). We refer to the estimated concentration of GABA as GABA+ to account for the contamination of co-edited macromolecules. The reported GABA+ concentration was water-scaled and alpha-corrected. GABA+ concentrations were normalized to the individual regional mean. The green bars represent the normalized GABA+ level obtained when signal was presented to the dominant eye (DE), and the purple bars represent the normalized GABA+ level obtained when signal was presented to the non-dominant eye (NDE). (a) Pre- and post-training normalized GABA+ concentrations in the early visual cortex cortex (EVC). (b) Pre- and post-training normalized GABA+ concentrations in the motor cortex (M1). Each connected dot pair in the figure corresponds to the normalized GABA+ concentrations measured from one participant when signal dots were presented to the dominant and the non-dominant eye. Error bars represent ±1 SEM. *p < 0.05.

The effects of perceptual training on normalized GABA+ concentrations and their relationship to SED were supported by a full 2 (Group—dichoptic/binocular)*×*2 (ROI—EVC/M1)×2 (Session—before/after training)×2 (Stimulus Configuration—signal dots presented to the dominant/non-dominant eye) mixed ANOVA that indicated a significant four-way Group×ROI×Session×Stimulus Configuration interaction (F_(1, 45)_= 7.978, p = 0.007, η^2^_p_= 0.153). This four-way interaction was followed up through separate corrected three-way mixed ANOVAs for each ROI. The analysis for the EVC voxel revealed a significant Group×Session×Stimulus Configuration interaction (F_(1, 45) = _7.107, p = 0.011, η^2^_p_= 0.139). The analysis for the M1 voxel, however, indicated no significant main effects or interactions (Fig. 4b).

To further explore the significant Group×Session×Stimulus Configuration interaction for the EVC voxel, we conducted separate, corrected mixed ANOVA for each of the dichoptic and binocular training groups (Fig. 4a). For the dichoptic training group, there was a significant interaction between Session and Stimulus Configuration (F_(1, 22)_= 8.763, p = 0.007, η^2^_p_= 0.294). Before training, GABA+ concentration was higher when signal dots were presented to the non-dominant eye (t_(22)_= -2.476, p = 0.021). After training, GABA+ concentration decreased when signal dots were presented to the non-dominant eye (t_(22)_= -2.96, p = 0.007) and increased when signal dots were presented to the dominant eye (t_(22)_= 2.96, p = 0.007). Notably, GABA+ concentration remained significantly different between the two stimulus configurations after training (t_(22)_= 2.815, p = 0.010) but in the direction that is opposite to the pre-training data: GABA+ level was higher when signal dots were presented to the dominant eye than to the non-dominant eye.

For the binocular training group, the Session×Stimulus Configuration mixed ANOVA revealed that GABA+ concentration differed between stimulus configurations (F_(1, 23)_= 17.193, p < 0.01 η^2^_p_= 0.439), but these differences did not vary with the scanning session (F_(1, 23)_= 0.391, p = 0.538). Upon concatenating the data across the two scanning sessions, we observed significantly higher GABA+ concentrations when the signal dots were presented to the non-dominant eye than when they were presented to the dominant eye (t_(23)_= -4.146, p < 0.01). The non-normalized GABA+ concentrations in the EVC and M1 are presented inFigure 5. The changes in the concentration of Glx (glutamate + glutamine) are depicted inFigure S3.

**Fig. 5.**
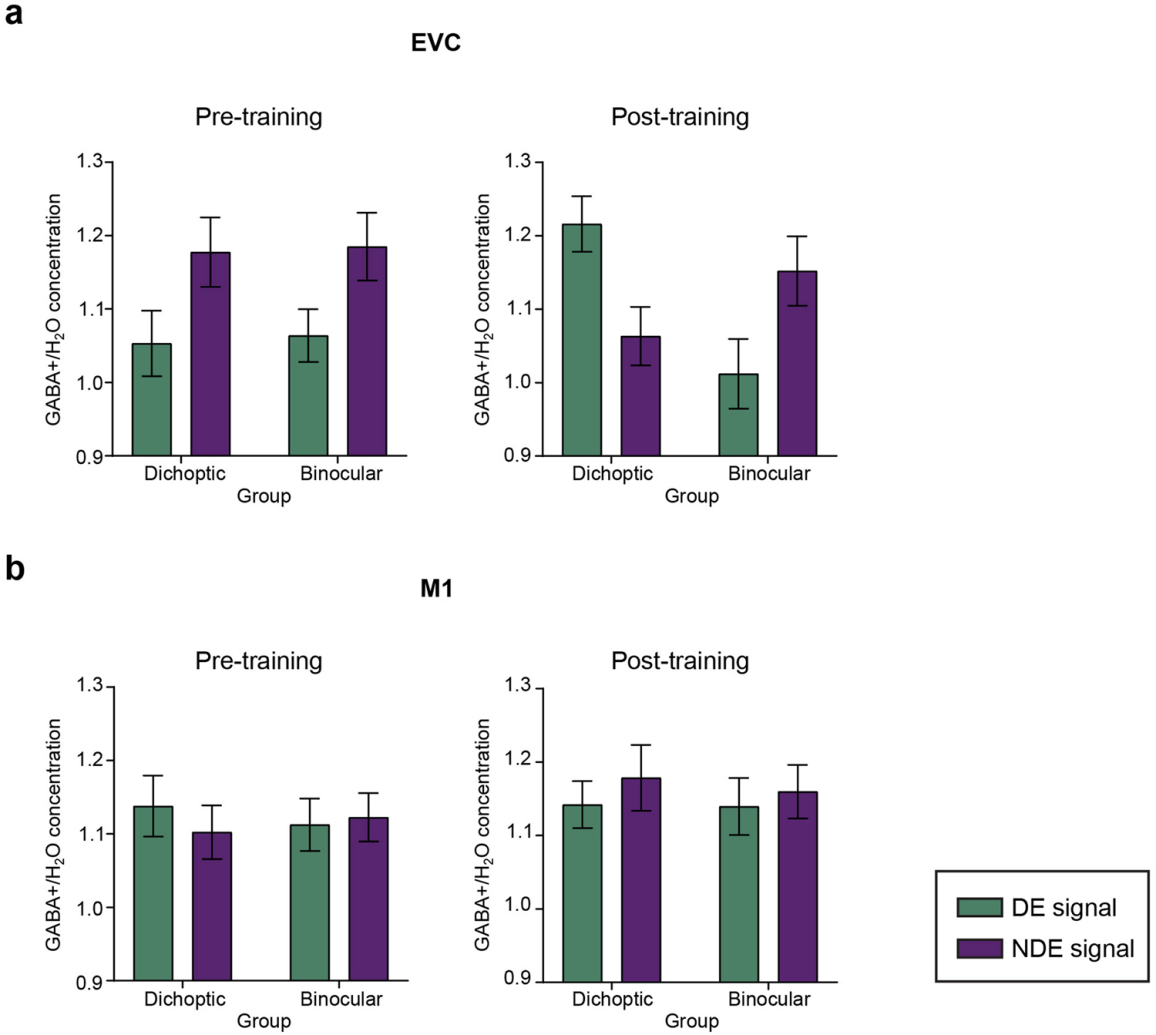
Pre- and post-training non-normalized GABA+ concentrations in (a) the early visual cortex (EVC) and (b) the motor cortex (M1). Error bars represent ±1 SEM.

Notably, the observed differences in GABA+ concentrations between the two stimulus configurations and their alterations following dichoptic perceptual training were not attributable to the differences in the noise levels between the two stimulus configurations. The stimulus values used in the bore were individually tailored using the thresholds obtained from a behavioral run completed inside the bore before MRS data acquisition. We can test this question of noise by extrapolating to comparisons of GABA concentrations before and after training, during which the overall stimulus SNR was notably different, and by comparing GABA concentrations between observers exposed to higher versus lower noise stimuli during MRS scans. To test for the between-session differences, we calculated the mean of unnormalized GABA+ concentration values in EVC for each scanning session and compared them using a paired t-test. The analysis indicated no significant differences in overall EVC GABA+ levels between scanning sessions despite noisier stimuli being shown at post-scan (t_(46)_= 0.283, p = 0.778). To compare GABA concentrations between observers who were presented with higher versus lower noise stimuli, we first divided the GABA data into two groups based on the motion thresholds obtained before MRS data acquisition. Specifically, observers with a motion threshold above the mean threshold were assigned to one group, while the other group included observers with a motion threshold below the mean. The overall unnormalized EVC GABA+ concentrations were then compared between the two groups. The analyses, however, did not reveal any significant differences in GABA+ concentrations between the two groups (pre-scan, t_(45)_= 1.545, p = 0.129; post-scan, t_(45)_= 0.129, p = 0.898). It is, therefore, unlikely that the differences in GABA concentration were driven simply by differences in the noise level of the motion stimulus between the two configurations.

### 3.3 Brain-behavior correlations

Finally, to further probe the functional relevance of GABA+ levels for SED and learning-induced changes, we conducted two sets of correlational analyses for comparing binocular balance indices and GABA+ concentrations. For each ROI, we first computed GABA+ concentration differences by subtracting the GABA+ measurements obtained during presentations of signals to the non-dominant eye from those obtained during presentations of signals to the dominant eye. A negative value here, thus, indicates higher GABA+ concentrations when signals are presented to the non-dominant eye. Any data points with a Cook’s distance larger than 4/n (where n is the total number of data points) were excluded from the correlational analyses.

**Fig. 6.**
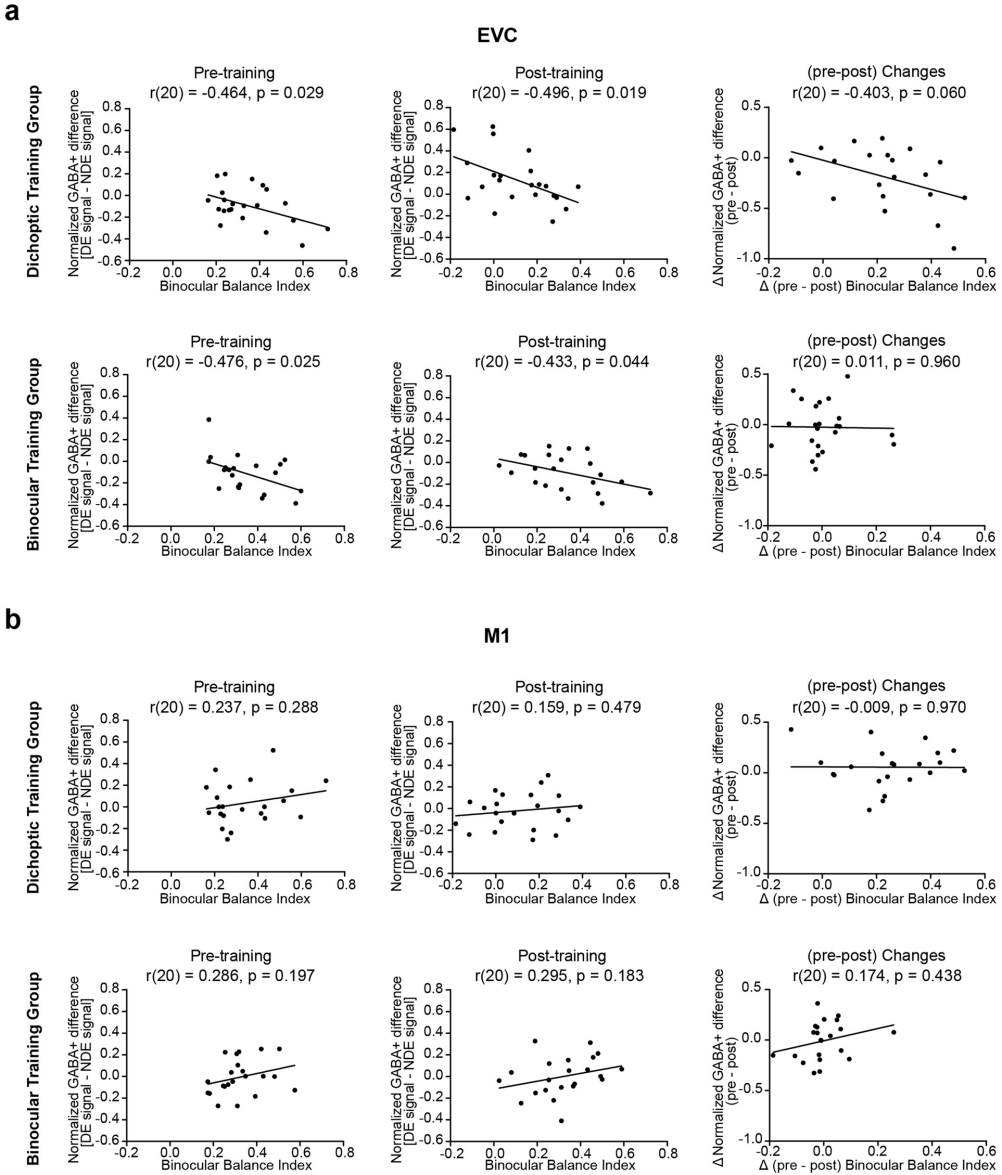
Correlations between behaviorally derived sensory eye dominance and metabolite concentrations of GABA+. (a) The left and middle columns display the correlations between GABA+ differences and binocular balance index in EVC before and after training, visualized independently for the dichoptic and binocular training groups. GABA+ concentration differences were computed by subtracting GABA+ measurement obtained during presentations of signals to the non-dominant eye from that obtained during presentations of signals to the dominant eye. The right column presents correlations between changes in GABA+ differences (pre−post) and changes in the binocular balance index (pre−post), displayed separately for the two training groups. (b) The left and middle columns show the correlations between GABA+ differences and binocular balance index in M1 before and after training, depicted independently for the two training groups. The right column presents correlations between changes in GABA+ differences and changes in the binocular balance index, presented separately for the two training groups.

First, we correlated GABA+ concentration differences in the EVC and M1 with binocular balance indices, separately for the pre- and post-training data (Fig. 6, left and middle columns). The analyses indicated a significant negative correlation between GABA+ concentration differences and binocular balance indices in the*EVC*for both groups prior to perceptual training (dichoptic, r_(20)_= -0.464, p = 0.029; binocular, r_(20)_= -0.476, p = 0.025). Specifically, individuals with stronger eye dominance tended to demonstrate lower GABA+ concentrations when their dominant eye received signals and higher GABA+ concentrations when their non-dominant eye received signals. After perceptual training, the negative correlations between GABA+ concentration differences and binocular balance indices remained for both groups in the EVC (dichoptic, r_(20)_= -0.496, p = 0.019; binocular, r_(20)_= -0.433, p = 0.044), although the correlation for the binocular training group did not survive statistical correction (q < 0.05). By contrast, we observed no correlations among any of the comparisons involving M1 in either group.

In the second set of correlational analyses, we examined the relationship between changes in GABA+ concentration difference (pre–post) and changes in SED (pre–post) following perceptual training. The results revealed that changes in GABA+ concentration difference in EVC trended toward a negative correlation with changes in SED for the*dichoptic*training group, as depicted inFigure 6a(right column), but did not reach statistical significance (r_(20)_= -0.406, p = 0.060). Specifically, individuals with greater changes in binocular balance index, indicating an improved balance after dichoptic training, tended to show a larger reduction in GABA+ concentration when signals were presented to their non-dominant eye and a larger increase in GABA+ concentration when signals were presented to their dominant eye after training. By contrast, no correlation was observed between changes in GABA+ concentrations in M1 and changes in SED for this training group (r_(20)_= -0.009, p = 0.970,Fig. 6b, right column). For the binocular training group, changes in SED did not correlate with changes in GABA+ levels in either the EVC (r_(20)_= 0.011, p = 0.960,Fig. 6a, right column) or M1 (r_(20)_= 0.174, p = 0.438,Fig. 6b, right column).

## 4 Discussion

We used MRS to examine the role of inhibitory mechanisms in the EVC, as measured by GABA concentrations, in mediating learning-driven SED plasticity. Specifically, we measured GABA concentrations in the EVC and M1 before and after a five-day perceptual training protocol and compared learning-related changes in behavior and metabolite concentrations between individuals trained on a dichoptic signal-in-noise motion task and those trained on a binocular variant of the same task. In terms of behavior, both training groups showed improvement in their respective training tasks; however, only the dichoptic training group demonstrated a significant shift in SED. MR spectroscopy data showed that before perceptual training, GABA+ concentrations in the EVC were lower when signals were presented to the dominant eye than when presented to the non-dominant eye, for both groups. After dichoptic perceptual training, GABA+ concentrations in the EVC increased during presentations of signals to the dominant eye and decreased during presentations of signals to the non-dominant eye, relative to pre-training baselines. These changes were not observed in the group trained on the binocular variant. Importantly, the observed associations between GABA+ levels and SED were specific to EVC, as GABA+ levels in the motor cortex were comparable when either eye received signals and did not change with either type of training.

We first discuss our behavioral findings. As in our previous fMRI work (Kam & Chang, 2023), we elected to contrast learning effects resulting from the dichoptic signal-in-noise motion training with those from training on a binocular variant of the same task. The rationale behind this comparison was twofold. First, to eliminate the possibility that any changes in SED following dichoptic training could be attributed to general training in motion perception. Second, and more importantly, previous research has demonstrated that GABAergic inhibition plays a critical role in perceptual learning (Heba et al., 2016;Lea-Carnall et al., 2020), particularly when trained with motion-in-noise stimuli (Frangou et al., 2018,^2019^). Therefore, it was important to include a second training task that was equivalently demanding to the main training task, that included the same motion-in-noise features, but yet did not require dichoptic integration. Our behavioral data are in good agreement with our previous fMRI work showing that while both variations of signal-in-noise motion training led to improved performance on the trained task, only the dichoptic training group showed a shift in SED after training (Kam & Chang, 2023). Importantly, learning effectiveness was comparable for both training groups in terms of percentage threshold changes and learning rate. As such, we consider the observed eye balance improvements to be genuinely attributed to the specific characteristics of dichoptic presentation and not a result of enhancements in motion perception or general enhancements in segregating signals from noise.

We next turn to MR spectroscopy data. To reveal the differences in inhibitory responses between the two eyes, we first compared GABA+ levels in the EVC during presentations of signals to the dominant and non-dominant eyes*before training*. Critically, our results indicated significant differences in GABA+ concentrations for both training groups, with more GABA+ when signals were presented to the non-dominant eye than to the dominant eye. At first glance, our data appear to conflict with previous findings byIp et al. (2020), who reported higher GABA levels in response to stimulation of the dominant eye and lower GABA levels in response to stimulation of the non-dominant eye. However, it is important to note that this previous study measured GABA concentrations during monocular stimulation, in which visual stimuli were presented to either the dominant or non-dominant eye with the other eye patched. It is possible that interocular inhibitory interactions observed during monocular stimulation differ from those observed during binocular or dichoptic stimulation.

We consider first a scenario in which the signal is presented to the*non-dominant*eye. In this case, two separate mechanisms may come into play. First, the non-dominant eye may expend considerable effort to enhance neuronal gain by suppressing data from the dominant eye (in this case, very noisy data), and/or counteracting the strong inhibition it receives from the dominant eye. Second, as a response to the increased gain attempt by the non-dominant eye, the dominant eye may also strive to suppress data from the non-dominant eye and compete for greater gain within the binocular network. We conjecture that these factors may contribute to a stronger overall inhibition and, hence, higher GABA+ concentrations. Conversely, when the signal is directed towards the*dominant*eye, less effort may be required to amplify the signal and suppress competing data from the non-dominant eye, given that the dominant eye already holds a dominant position. As a result, the non-dominant eye may also experience decreased engagement in competitive processes. This reduced demand for signal enhancement and competition ultimately leads to a lower level of inhibition and, thus, lower GABA+ concentrations. Despite several MRS studies having established clear correlations between behavioral measures of binocular rivalry and GABA concentrations measured at rest (Pitchaimuthu et al., 2017) or during monocular or binocular presentations of visual stimuli (Ip et al., 2020;Robertson et al., 2016), there is a notable lack of work that attempts to measure GABA concentrations during conditions that permit speculation on interocular inhibitory interactions (i.e., during systematic manipulations of dichoptically presented information). It is, therefore, challenging to compare our observations to the existing literature. Nonetheless, the interpretation of our results remains speculative, and further empirical verification will be required moving forward.

We next turn our attention to the changes in GABA+ concentrations*following perceptual training*. The only existing study that examined GABA-ergic changes as they relate to SED plasticity reported decreased resting GABA concentrations after short-term monocular deprivation, but no significant GABA alterations were observed during either monocular or binocular viewing conditions (Lunghi et al., 2015). Here, we did not observe an overall reduction in GABA+ levels after dichoptic perceptual training. Instead, we observed differential alterations in GABA+ levels depending on whether signals were presented to the dominant or non-dominant eye. Specifically, we found that after dichoptic (but not binocular) perceptual training, GABA+ concentrations in the EVC significantly increased during presentations of signals to the dominant eye and reduced during presentations of signals to the non-dominant eye, relative to pre-training levels. The discrepancy between our current findings and those reported previously can be attributed to two key factors: the different paradigms employed to induce SED plasticity (short-term monocular deprivation versus longer-term visual training), and the variations in visual stimulation during the measurements of GABA concentration. Specifically, unlike our current approach, which involves systematic manipulations of dichoptically presented information during MRS data acquisition,Lunghi et al. (2015)employed a paradigm in which participants viewed luminance-modulated checkerboards on a screen with either one eye viewing while the other eye was occluded or with both eyes viewing simultaneously. Notably, measuring GABA concentrations during these viewing conditions could reflect interocular inhibitory interactions that differ from those observed during dichoptic presentation.

Drawing upon our understanding of the inhibitory circuits involved in binocular combination (Meese et al., 2006), as well as the established association between GABA levels and interocular inhibition (Mentch et al., 2019;Robertson et al., 2016;Sengpiel, 2005;Sengpiel et al., 2006;van Loon et al., 2013), the observed alterations in GABA+ concentration within this study indicate a potential readjustment or rebalancing of interocular inhibition in the EVC. This rebalancing of interocular inhibition may involve a possible weakening of inhibition originating from the dominant eye and/or a strengthening of inhibition arising from the non-dominant eye. In the context where the signal is presented to the*non-dominant*eye, it is possible that less effort (compared to pre-training) is now required for the non-dominant eye to enhance neuronal gain and counteract the inhibitory effects exerted by the dominant eye as the inhibition originating from the dominant eye may reduce after dichoptic training. Consequently, the overall interocular inhibition is diminished, leading to a corresponding decrease in GABA+ concentrations under these configurations. By contrast, when the signal is presented to the*dominant*eye, despite its continued dominance (in most cases), the inhibitory signals from the non-dominant eye may have strengthened after dichoptic training. Therefore, the dominant eye is compelled to exert heightened effort in order to suppress the competing data originating from the non-dominant eye. This simultaneous increase in the strength of inhibition exerted by both eyes may explain the observed increase in GABA+ concentrations. Despite this speculative interpretation, it is evident from our data that dichoptic training alters the dynamics of interocular inhibition in the EVC, with reduced inhibition during presentations of signals to the non-dominant eye and increased inhibition during presentations of signals to the dominant eye.

Following the above, if the reduction in SED is driven by a rebalancing of interocular inhibition, we might have expected for GABA+ levels during presentations of signals to the dominant eye to become comparable to those observed during presentations of signals to the non-dominant eye. However, our findings are somewhat surprising. Despite significant changes in GABA+ concentrations (relative to pre-training) for both stimulus configurations, GABA+ concentrations measured*between*the two stimulus configurations remained significantly different after dichoptic training. Strikingly, the direction of this difference (between the two stimulus configurations) was opposite to the pre-training data, with higher GABA+ concentrations observed when signals were presented to the dominant eye compared to the non-dominant eye. Upon examination of the post-training data, we speculate that the observed reversed trend in GABA+ concentrations after dichoptic training may be attributed to a subgroup of individuals whose dominant eye not only became less dominant after training, but even became slightly weaker than the other eye.

**Fig. 7.**
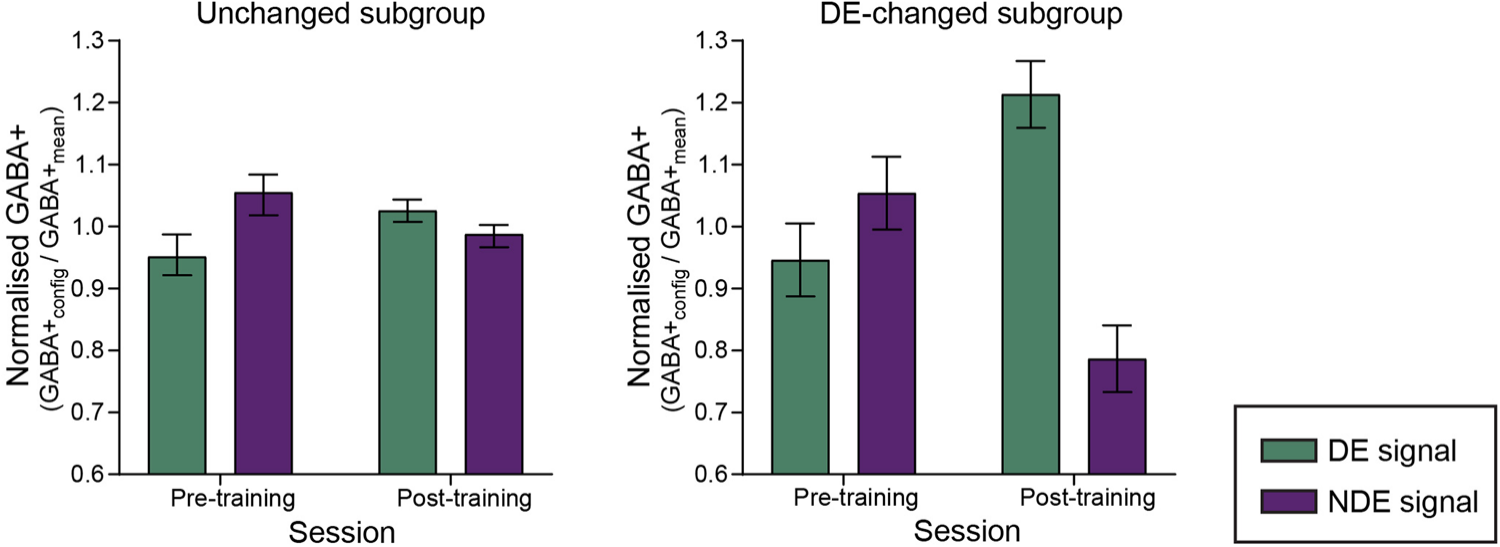
Pre- and post-training GABA+ data for the two subgroups (unchanged and DE-changed subgroup) of the dichoptic training group. We identified five observers in the dichoptic training group whose dominant eye changed after training. The green bars represent the normalized GABA+ level obtained when the signal was presented to the dominant eye (DE), and the purple bars represent the normalized GABA+ level obtained when the signal was presented to the non-dominant eye (NDE). Error bars represent ±1 SEM.

In line with our previous fMRI work (Kam & Chang, 2023), 20% of the observers in the dichoptic training group exhibited a behavioral change in the dominant eye (indicated by a sign change of the binocular balance index) after training.Figure 7displays the breakdown of the pre- and post-training GABA data separately for the “DE-changed” and “unchanged” subgroups. By examining the data for each subgroup, we observed that while the dichoptic training group, as a whole, demonstrated significant changes in GABA+ concentrations for both stimulus configurations after training (as per the analyses reported in theResultssection), the two subgroups exhibited different magnitudes of GABA+ changes. Remarkably, the DE-changed subgroup exhibited noticeably larger changes in GABA+ concentrations for both stimulus configurations after training. Compared to pre-training levels, post-training GABA+ concentrations appeared to be considerably higher when signals were presented to the dominant eye and lower when signals were presented to the non-dominant eye. Notably, there were also apparent differences in post-training GABA+ concentrations*between*the two stimulus configurations. The unchanged subgroup, on the other hand, despite showing the same pattern of GABA+ level changes as the DE-changed subgroup (an increase when signals were presented to the dominant eye and a decrease when signals were presented to the non-dominant eye, relative to pre-training GABA+ level), tended to display comparatively smaller changes in GABA+ concentrations for both stimulus configurations after training. Further, GABA+ levels*between*the two stimulus configurations appeared more balanced after training for this subgroup. Importantly, the DE-changed subgroup demonstrated not only larger changes in GABA+ levels, but also exhibited greater changes in SED when compared to the unchanged subgroup (Fig. S2). This observation indeed aligns with our correlational analysis, which indicated that individuals who experienced greater changes in SED tended to exhibit a larger reduction in GABA+ concentration when signals were presented to their non-dominant eye, and a corresponding larger increase in GABA+ concentration when signals were presented to their dominant eye. These findings suggest that dichoptic training, in general, may work by promoting a rebalancing of interocular inhibition in the EVC. It is noteworthy, however, that within the DE-changed subgroup that experienced comparatively larger changes in SED, interocular inhibition surpassed mere rebalancing and instead became more exaggerated.

While our findings provide insight into the GABA-mediated inhibitory mechanisms underlying learning-driven SED plasticity in adults, we note that this study has a major limitation that merits discussion. Specifically, the spatial resolution of our MRS voxel did not permit precise targeting of a single visual area. The EVC voxel was primarily positioned to target bilateral V1; however, portions of V2 and V3 were also included in our measurements. As discussed, learning-driven eye balance changes could be driven by mechanisms before and/or after binocular summation (Meese et al., 2006). Animal physiological studies have shown that interocular suppression of visual responses occurs not only in V1 but also in V2 (Bi et al., 2011;Shooner et al., 2017). Additionally, an fMRI study demonstrated that short-term monocular deprivation in healthy adults modulates activity beyond V1 (Binda et al., 2018). Therefore, we cannot rule out the possibility that inhibitory mechanisms taking effect in visual areas beyond V1 may have also contributed to our results. Opting for a smaller voxel size enhances spatial resolution but reduces signal-to-noise ratio due to smaller volume of tissue being examined. Moreover, even when confining the voxel to include only V1, it remains challenging to determine whether the observed changes were driven by pre-summation or post-summation interocular inhibitory mechanisms. This ambiguity arises as changes could occur in the input layer, where signals from the two eyes remain segregated, or in the upper layers, after binocular combination. In V1, parvalbumin (PV) and somatostatin interneurons are the primary types of GABAergic neurons (Kelly et al., 2019;Rudy et al., 2011;Van Brederode et al., 1990). PV neurons are particularly important for early ocular dominance plasticity (Gu et al., 2016;Sun et al., 2016;Tang et al., 2014), and the perineuronal nets surrounding PV interneurons play a critical role in governing ocular dominance plasticity in adult rats (Boggio et al., 2019). Hence, the variation in GABA levels observed during presentations of signals to the dominant and non-dominant eye, as well as the learning-related changes identified in this study, may signify the involvement of PV interneurons and their inhibitory effects. Rodent studies have revealed that PV interneurons within V1 are abundant not only in the input layer, but also in higher layers where binocularity is established (Gonchar & Burkhalter, 1997;Gonchar et al., 2008). The current data, therefore, are insufficient to draw conclusions regarding whether dichoptic training acts on inhibitory mechanisms before and/or after binocular combination.

## 5 Conclusion

The present data reveal that interocular inhibitory mechanisms at the EVC play a pivotal role in mediating SED. Dichoptic perceptual training shifts inhibition patterns in the EVC, characterized by increased inhibition during presentations of signals to the dominant eye and reduced inhibition during presentations of signals to the non-dominant eye. Our findings suggest that dichoptic perceptual training drives changes in SED by potentially promoting a rebalancing of interocular inhibition in the EVC. These findings enhance our understanding of the neural mechanisms underlying learning-driven SED plasticity and, therefore, may serve as a foundation for the advancement of region-specific therapeutic approaches for individuals with visual impairment, particularly for those with clinical binocular imbalance.

## Supplementary Material

Supplementary Material

## Data Availability

The data that support the findings of this study are available from the corresponding author upon reasonable request, subject to additional approval from the Human Research Ethics Committee of The University of Hong Kong. The data are not publicly available due to their inclusion of sensitive information that could potentially compromise the privacy of research participants. Custom code written for the presentation of stimuli in this manuscript is available from the corresponding author upon reasonable request.
