# Supplementary material for: The neurochemistry of learning-driven sensory eye dominance plasticity
Source: Imaging Neurosci (Camb). 2024 Jul 22;2:imag-2-00237. doi: 10.1162/imag_a_00237 (PMC12272184; doi:10.1162/imag_a_00237)
Supplement: Supplementary Material [file imag_a_00237-supp.pdf]

Supplementary materials for
The Neurochemistry of Learning-driven Sensory Eye Dominance Plasticity

This PDF file includes:

Figure S1
Figure S2
Figure S3
Figure S4
Table S1

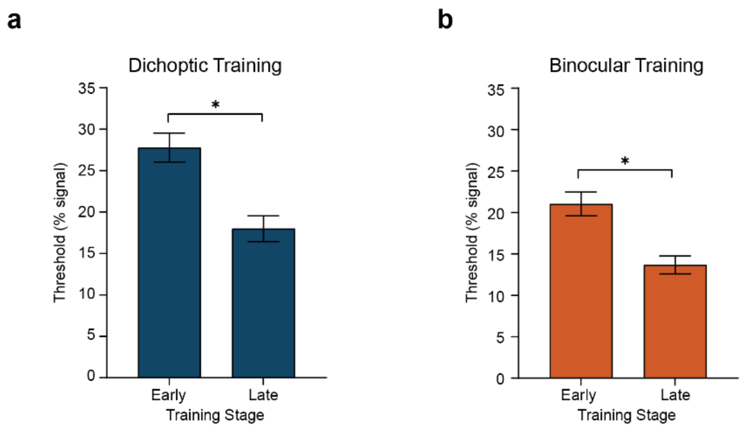

Figure S1. Early and late training thresholds for the (a) dichoptic (N = 23) and (b) binocular (N = 24) training groups. The early and late training thresholds were calculated by averaging the first and the last three training blocks, respectively.

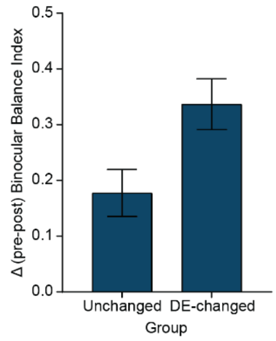

Figure S2. Changes in binocular balance index (pre – post) for the two subgroups (unchanged and DE-changed subgroup) of the dichoptic training group. Error bars represent ±1 SEM.

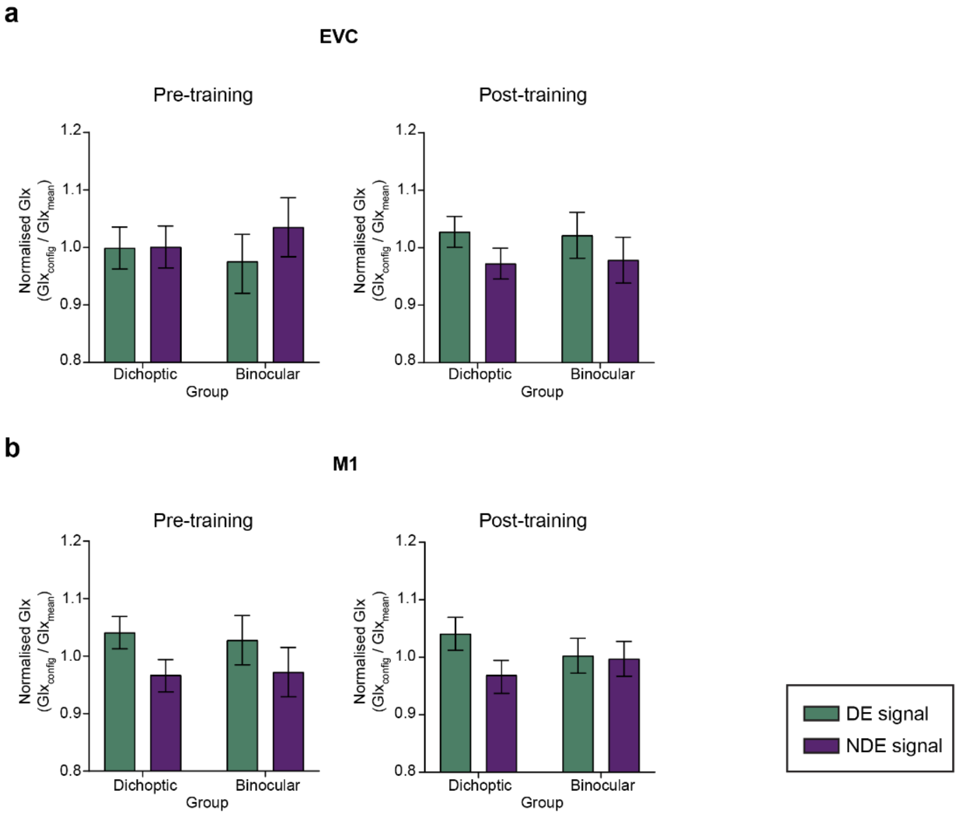

Figure S3. **(a)** Pre- and post-training normalized Glx (glutamate + glutamine) concentrations in the early visual cortex (EVC). **(b)** Pre- and post-training normalized Glx concentrations in the motor cortex (M1). To examine the specificity of learning-induced metabolite changes, we extracted the concentration of Glx, and conducted a 2 (Group – dichoptic/binocular) × 2 (ROI – EVC/M1) × 2 (Session – before/after training) × 2 (Stimulus Configuration – signal dots presented to the dominant/non-dominant eye) mixed ANOVA. The analysis revealed no significant main effects or interactions, indicating that there were no variations in Glx concentrations for conditions where the signal was presented to the dominant eye compared to the non-dominant eye, in addition to no learning-related changes in Glx. Error bars represent ± 1 SEM.

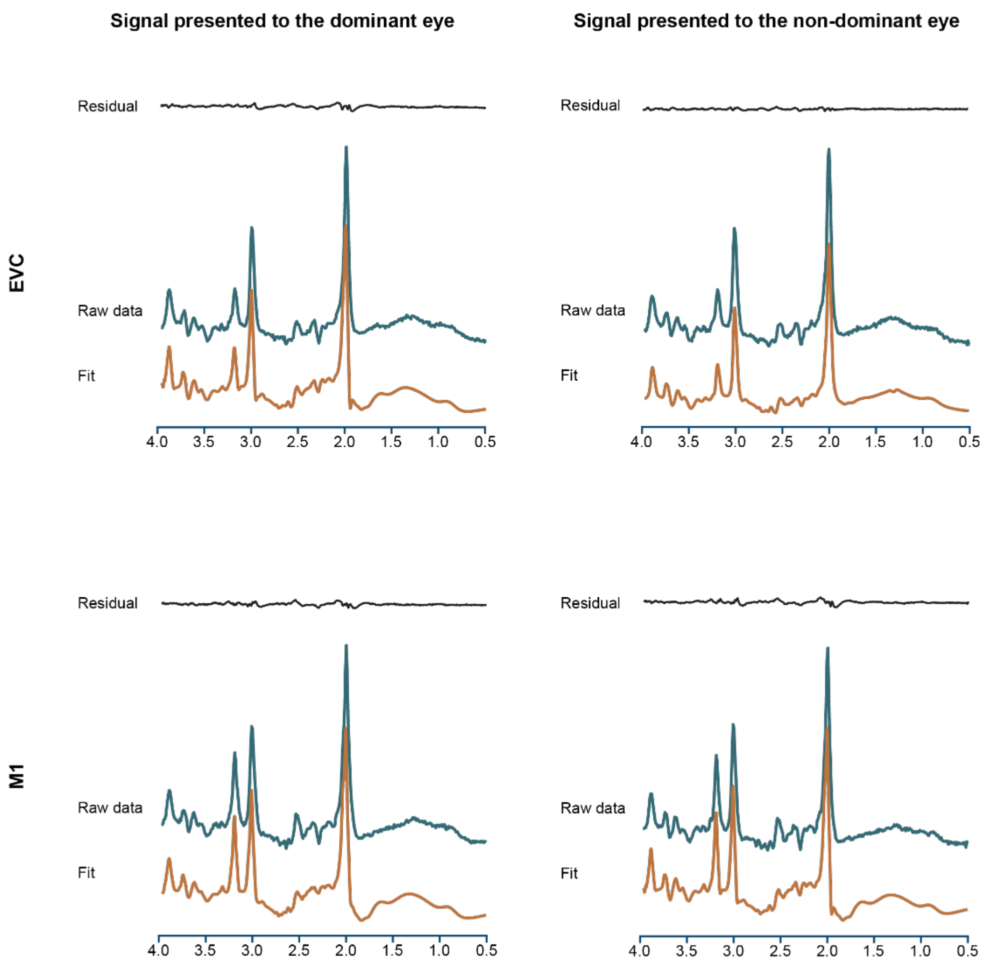

Figure S4. Example spectra obtained from the early visual cortex (EVC) and the motor cortex (M1) voxel for one participant, presented separately for the two stimulus configurations (signal presented to the dominant versus the non-dominant eye). The blue spectra show the raw data, while the orange spectra display the fitted data. The black spectra depict the residuals resulting from the model fit.

Table S1.

The mean Talairach and MNI coordinates of the voxel's centroid for early visual cortex (EVC), left motor cortex (M1) and right M1, presented independently for pre-training and post-training MRS.

		X	Y	Z	X	Y	Z
		Pre-training			Post-training		
				EVC			
Dichoptic	TAL	0.90±1.81	-73.16±2.52	0.92±2.97	0.93±1.97	-73.36±2.44	1.10±3.06
	MNI	0.0±2.15	-74.46±2.52	-4±3.30	0.29±2.01	-74.79±2.34	-3.92±3.43
Binocular	TAL	0.73±1.84	-72.79±2.83	0.72±3.24	0.91±1.48	-72.87±2.76	0.94±2.76
	MNI	0.12±1.99	-74.32±2.67	-4.32±3.94	0.32±1.55	-74.60±2.78	-3.88±3.70
				Left M1			
Dichoptic	TAL	-20.24±1.63	-21.64±3.05	43.91±2.50	-20.25±1.46	-21.54±3.01	44.25±1.59
	MNI	-20.20±1.93	-24.20±3.19	47.10±3.14	-19.90±1.52	-23.90±3.35	47.40±2.22
Binocular	TAL	-19.93±1.78	-22.36±4.73	44.54±1.77	-19.47±1.99	-22.11±4.21	44.74±1.72
	MNI	-19.54±1.81	-24.69±4.75	47.69±2.21	-19.00±1.87	-24.46±4.37	48.23±1.96
				Right M1			

Dichoptic	TAL	21.43±1.97	-23.07±1.90	44.86±1.84		21.28±1.51	-22.87±1.87	45.36±2.13
	MNI	21.17±2012	-25.58±1.97	47.42±2.27		20.92±1.83	-25.20±2.15	48.42±2.68
Binocular	TAL	22.33±1.20	-22.91±3.12	45.0±2.57		21.78±1.23	-22.27±3.61	45.59±2.99
	MNI	21.70 ±1.16	-25.60±3.10	48.00±3.06		21.00±1.33	-25.10±3.76	48.80±3.61
